# Colorectal Cancer Stem Cells in the Progression to Liver Metastasis

**DOI:** 10.3389/fonc.2020.01511

**Published:** 2020-08-20

**Authors:** Carlos A. Gonzalez-Villarreal, Adriana G. Quiroz-Reyes, Jose F. Islas, Elsa N. Garza-Treviño

**Affiliations:** ^1^Universidad de Monterrey, UDEM Vicerrectoria de Ciencias de la Salud, San Pedro Garza García, Mexico; ^2^Universidad Autonoma de Nuevo Leon Facultad de Medicina, Departamento de Bioquimica y Medicina Molecular, San Nicolás de los Garza, Mexico

**Keywords:** cancer stem cells, liver metastasis, epithelial–mesenchymal transition, pericytes, circulating tumor cells, metastasis, colorectal cancer

## Abstract

Colorectal carcinoma (CRC) is a leading cause of cancer mortality. Tumorigenesis is a dynamic process wherein cancer stem cells (CSCs) and their microenvironment promote initiation, progression, and metastasis. Metastatic colonization is an inefficient process that is very complex and is poorly understood; however, in most cases, metastatic disease is not curable, and resistance mechanisms tend to develop against conventional treatments. An understanding of the underlying mechanisms and factors that contribute to the development of metastasis in CRC can aid in the search for specific therapeutic targets for improving standard treatments. In this review, we summarize current knowledge regarding tumor biology and the use of stroma cells as prognostic factors and inflammatory inducers associated with the use of tumor microenvironments as a promoter of cancer metastasis. Moreover, we look into the importance of CSC, pericytes, and circulating tumor cells as mechanisms that lead to liver metastasis, and we also focus on the cellular and molecular pathways that modulate and regulate epithelial–mesenchymal transition. Finally, we discuss a novel therapeutic target that can potentially eliminate CSCs as a CRC treatment.

## Introduction

Colorectal carcinoma (CRC) is one of the most common tumors, with a global occurrence of over 2 million new cases each year (1). CRC represents the third most common cancer worldwide in terms of incidence and mortality for both men and women (2). Approximately 20–25% of patients with CRC already exhibit metastases at the time of diagnosis, and a striking 25% will develop metastatic spread during or after their follow-up (3). Briefly, CRC metastases begin with a series of mutations in the epithelial cells of the colon, continuing with the detachment of several cells from the primary tumor. This is followed by the invasion of surrounding tissue, diffusion through blood or lymphatic circulation, establishment, and finally, their proliferation to a distant location (1, 2).

Common sites of distant metastasis include the liver with a frequency of 60% and 25% to the peritoneum (3). Furthermore, the total number of metastases, tumor volume, and tumor microenvironment (TME) composition are strong predictors of prognosis (4). TME regulation is highly dependent on exosome production. Exosomes are extracellular vesicles (30–150 nm in diameter) that carry various contents such as growth factors, miRNAs, and enzymes. Sites of interactions between cancer cells and their microenvironment include blood vessels, extracellular matrix (ECM), signaling molecules such as transforming growth factor-beta (TGFβ), angiopoietin 1 and 2, platelet-derived growth factor (PDGF), and vascular endothelial growth factor (VEGF). They also include other tumor-recruited cells such as cancer-associated fibroblasts (CAFs), stromal myofibroblasts, endothelial cells, pericytes, diverse immune cells, mesenchymal stem cells, and cancer stem cells (CSC). All of these contribute to tumor growth and serve as a prerequisite for tumor cell invasion and metastasis (5). Table 1 describes the participation of each cell type in the development or establishment of metastasis.

**Table 1:** TME-associated cells.

**Cell type**	**Identification markers**	**Normal activity**	**Prometastatic activity**	**References**
CSC	Nanog Oct-4 SOX-2 Lgr-5 CD133 CD24 CD29 ALDH1 EpCAM CD44 CD166 CD26	Not present in healthy tissues	Tumor development, chemoattractant activity (SDF-1/CXCL12)	(6)
CAFs	CD44 a-SMA PDGFR-b Desmin FAP FSP MFAP5 TN-C PDPN NG2	Extracellular matrix synthesis of collagen and elastin.	Secretion of growth factors EGF, IL-6, ILGF, HGF, improve angiogenesis, and remodel of ECM	(7–12)
MSCs	CD105 CD73 CD44 CD90	Components of stroma, immunomodulatory functions	Improve cell proliferation, angiogenesis, and metastasis by the secretion of CXCL12, IL-6, IL-8, and differentiation in CAFs	(11, 13, 14)
Pericytes	PDGFRβ CD13 NG2 CSPG4 a-SMA Desmin RGS5 CFTR/MRP SUR2 ALP Vimentin CD133 CD146.	Protection of endothelial cells in capillaries, blood flow regulation, and inflammatory cell trafficking	Secretion of growth factors HGF, FGFs, and CXCL12 that promote the growth, survival of malignant cells, and function as a chemoattractant	(5, 15–17)
Tregs	CD4 CD25 FoxP3 CD127	Maintenance of immune tolerance	Inactivation of CD8+ T-cells' cytotoxic activities, improvement of angiogenesis, and myeloid cell recruitment. Bad prognostic indicator	(11, 18–20)
Tumor-associated macrophages (TAM) -M1 -M2	CD14, CD16, CD64, CD68, CD71 CCR5	Proinflammatory cells specialized in pathogen destruction and enhancing activation of cytotoxic lymphocytes Anti-inflammatory cells that stimulate a CD4+ and regulatory T-cell response	Elimination of malignant cells. High production of NO TNFα, CXCL9, and CXCL10 Activate secretion of IL-6, TGFβ, EGF, bFGF, and IL-10, and VEGF to enhance tumor growth	(3, 11)

CSCs, also known as tumor-initiating cells, have recently been described as responsible for tumor growth, relapse, and treatment resistance. These cells represent a small subset of tumor cells (1–10%) that are involved in tumor development and self-renewal (6). They are crucial in maintaining stemness, tumor cell proliferation, and differentiation, and they are known to be regulated by pluripotency-related transcription factors such as OCT4, SOX2, KLF4, MYC, and NANOG. Moreover, these cells have been characterized and are known to present the following markers: CD44, CD24, CD133, CD166, LGR5, ALDH1, CD29, CD26, and CD51 (6, 21), all of which are not exclusive markers. When either CRC or CSC presents the CD51 marker and is co-localized with the TGFβ receptor, they can promote TGFβ/Smad signaling that upregulates epithelial–mesenchymal transition (EMT)-related genes, such as PAI1, MMP9, and Snail, which then promotes sphere formation, cell motility, and subsequent tumor formation (22, 23). Moreover, the standard form of CD44 (CD44s) is encoded by 10 constant exons. Alternative splicing generates CD44 variant isoforms (CD44v), of which specific isoforms are key in tumor development. Previously, studies on tissue CRC patients have shown that CSC-expressing CD44v6 permitted migration, enhanced autophagy flux, caused the phosphorylation of AKT and ERKs in the presence of a chemotherapy drug, and generated metastatic tumors; these were all associated with poor prognosis (24). Furthermore, the microenvironment surrounding CSCs plays an important role in supporting EMT induction, enhancing migration, homing, recolonization, and establishing metastasis (5).

## Epithelial–Mesenchymal Transition

EMT is a phenotype switch carried out by epithelial cells that enables them to assume a mesenchymal phenotype, thereby enhancing their migratory capacity, among other things (23). This process improves CSC proliferation and allows for the invasion of other tissues, thereby establishing metastasis. During EMT, metastasis begins with the loss of cell–cell junctions (based on cadherins, claudins, and occludins), secretion of enzymes (e.g., matrix metalloproteinases), and the loss of apicobasal polarity due to the weak expression of cadherin–catenin anchorage to the cytoskeleton (25). It is worth noting that cadherins are among the cell adhesion molecules (CAMs) that include selectins, integrins, and proteoglycans. CAMs govern the assembly of cells into three-dimensional tissues, allowing epithelial cells to change to a mesenchymal phenotype, thus giving cancer cells acquired capabilities to extensively proliferate, invade, migrate, and metastasize (26). The overall mechanism of EMT can be regulated in several ways such as through epigenetic regulation, growth factors, and transcription factors (27). Tight regulation of EMT permits the timely expression of important biomarkers such as N-cadherin, vimentin, smooth muscle actin alpha (α-SMA), fibronectin, and other crucial regulatory factors such as the paired-related homeobox 1 (Prrx1) (23). Prrx1 functions in attenuating the complementary expression of Snail1, and the loss of Prxx1 is required for CSC to colonize organs *in vivo*, which in turn reverts them to an epithelial state and removes the acquired CSC phenotype (28).

**Figure 1:**
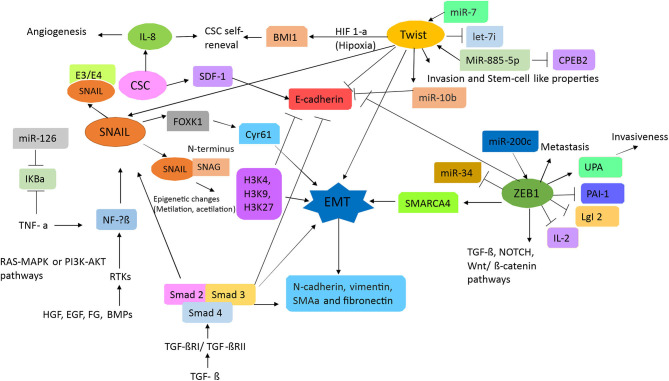
Schematic of the signal transduction pathways associated with epithelial–mesenchymal transition (EMT). TGFβ and RTK ligands mediate signaling in liver cells, playing pivotal roles as upstream mediators for inflammation, growth, cytoskeletal rearrangement, proliferation, reducing cell–cell adhesion, and metastasis. TGFβ induces the Smad signaling cascade to block E-cadherin, which is a strong blocker of EMT transition. Additionally, both TGF and RTKs can mediate inflammation by inducing SNAL/SNAG and by activating FOXk1 and Cyr61. Hence, inducing EMT via epigenetic changes results in the activation of α-SMA, N-cadherin, vimentin, and fibronectin. Stem cell signature is also further affected as miR-7 works in concert with Twist to block let-7 and activate miR-10b. Because of inflammatory loopback signaling, HIF-1a, along with IL-8, is no longer induced. Through this response, miR-34 is downregulated and miR-200c is upregulated, leading to the activation of ZEB1 (activator of EMT), which then upregulates SMARC4, setting up the pathway for metastasis. Furthermore, ZEB1 activation is responsible for TGFβ, NOTCH, and Wnt/β-catenin pathway activation, which then leads to proliferation.

Transcription factors are proteins that enable the transcription of genes by binding to specific sites in the promoter region of a gene. Hence, in concert, they induce the activation of signals responsible for the regulation of proliferation, invasion, metastasis, differentiation, therapy resistance, and apoptosis of cells (29). As such, EMT progression requires the transcription factors Snail1 (Snail), Snail2 (Slug), Snail 3 (Smuc), basic helix-loop-helix factors (TWIST1/TWIST2), and zinc finger E-box binding homeobox (ZEB) (23, 25). EMT activation through transcription factors is illustrated in Figure 1.

The transcription factor Snail is promoted by upstream signaling of the hepatocyte growth factor, epidermal growth factor (EGF), fibroblast growth factor (FGF), and bone morphogenic protein, resulting in downstream binding to receptor tyrosine kinases (RTKs). Furthermore, both the RAS-MAPK and the PI3K-Akt pathways can also activate NF-kβ, which consequently leads to Snail activation (30). An important trait of Snail is the SNAG domain in its N-terminus, which can recruit multiple chromatin enzymes such as HDAC, G9a, and Suv39H1 to the E-cadherin promoter. Chromatin modifications such as methylation at H3K27 can inhibit E-cadherin promoter activity (31). Moreover, Snail activates IL-8 expression by binding to its E3/E4 boxes, thus inducing cancer stem-like activities (32). Certain cytokines, such as tumor necrosis factor-alpha (TNFα), can induce the degradation of IκBα, which is an inhibitor of NF-kβ, and induce its nuclear translocation (33). This then induces the overexpression of Snail and other related genes (such as JUN) and correlates with both tumor and EMT progression (31).

As mentioned in other studies, TGFβ signaling plays an important role in EMT (30). TGFβ activates the Smad signaling pathway by binding to its receptors TGFβRI/TGFβRII, after which downstream effectors Smad2/Smad3 become phosphorylated. These then form a complex with Smad4, which induces the transcription of Snail1, vimentin, and fibronectin in CRC (31). Moreover, TGFβ signaling represses E-cadherin expression, which then initiates EMT (23). Furthermore, Snail1 activation also triggers FOXK1 expression, which is another EMT inducer, by upregulating the cysteine-rich angiogenic inducer 61 (Cyr61), which is a marker associated with metastasis by expression in CRC tissue (34).

The basic helix–loop–helix factor, TWIST, has an important role in metastasis, angiogenesis, chromosomal instability, and therapy resistance of tumors (30). Activation of its signaling pathway, along with Slug, leads to E-cadherin repression. Moreover, under hypoxic conditions, TWIST can regulate expression of the polycomb group RING finger protein BMI1 (BMI1) and promote the self-renewal of stem cells (31). The transcription factor TWIST-1 has also been reported to induce miR-10b, which promotes metastasis by targeting Homeobox D10 (HOXD10), which is associated with the inhibition of cell migration and may reduce E-cadherin expression, resulting in EMT (35); this mechanism is associated with hypermethylation of the miR-10 promoter in CRC tissues; however, studies with greater samples and in different stages to know the role in CRC progression were necessary. In a recent study, miR-10b was found to target Kruppel Like Factor 4 (KLF4), resulting in a classically metastatic and aggressive phenotype (36), but the molecular mechanism in CRC is not known.

The zinc finger E-box binding homeobox 1 (ZEB1) protein is the main element in EMT (37). ZEB1 can regulate expression of E-cadherin, vimentin, and various metalloproteases (25, 31). In CRC cells, ZEB1 can repress lethal giant larvae homolog 2 (LgI2) and promote metastasis (38). ZEB1 also contributes to the epigenetic silencing of E-cadherin and downregulates miR-34a expression. miR-34a helps remodel the cytoskeleton and improves the invasion and metastatic capabilities of CRC and colon CSC (39, 40). The invasiveness of a tumor can also be promoted by ZEB1 through the inhibition of plasminogen activator inhibitor-1 (PAI-1) and the activation of plasminogen activator (uPA); these are done by reducing its mRNA stability and through a mechanism involving histone acetyltransferase p300, respectively (31).

ZEB1 regulates other signaling pathways including TGFβ, Nanog, Notch, and canonical Wnt (β-catenin/TCF4) (41); this makes ZEB1 an ideal therapeutic target that can affect the population of CSC. Currently, there is no exclusive CSC marker nor an identified resistance mechanism that makes these cells less susceptible to conventional treatments. The miR200 family consisting of miR-200a, miR-200b, miR-141, and miR-429 (which all share a consensus seed sequence) have been gathering much attention, as they have been identified as key regulators of EMT, and are involved in reverse transition (23, 41). Other miRs can regulate cancer-related genes such as oncogenes or genes implicated in cell growth, cell survival, angiogenesis, and tissue differentiation. It was found that a miR-181a gene target, WiF-1 (Wnt inhibitory factor 1), leads to a potent liver metastasis effect by suppressing epithelial markers such as E-cadherin and β-catenin and by overexpressing the mesenchymal marker vimentin found in CRC tumor tissues with liver metastasis (42). In contrast, miR-19a has been stated to promote proliferation and invasion by targeting T-cell intracellular antigen-1 (TIA1), which is an important tumor suppressor reported in colon cancer cells (*in vitro*) and in a xenografted mice model (*in vivo*). Conversely, it has also been reported to inhibit tumorigenesis; this occurs through angiogenesis inhibition as a result of KRAS targeting (43). miR-17-92a is an exosomal miR cluster widely regarded as a protumoral miR (44), whereas miR-885-5p is overexpressed during CRC liver metastasis by inhibiting the cytoplasmic polyadenylation element-binding protein 2 (CPEB2), resulting in TWIST upregulation, which then leads to EMT. Both are detected in serum and CRC tissue but this requires validation with a greater number of samples.

## Cancer Stem Cells and Circulating Tumor Cells

As previously stated, epithelial tumor cells gain invasiveness and migratory abilities in the process of EMT; these are essential for successful metastatic spread and CSC generation (45). Diverse CSC clones can typically coexist within primary tumors, allowing for great intratumoral heterogeneity. After EMT, other CSC subpopulations may acquire properties such as quiescence, self-renewal, asymmetric division, drug resistance, and radiation resistance (46). Theoretically, under differing TME conditions, the CSC and non-CSC subpopulations undergo a dynamic conversion through EMT. Adversely, CSC can undergo the reverse mesenchymal–epithelial (MET) transition, which generates epithelial cells. These newly developed epithelial cells can be further extravasated into distant organs. It is known that disseminated cancer cells need to regain their epithelial phenotype to initiate the growth of a solid tumor at a secondary site (47).

Circulating tumor cells (CTC) are cells that have separated from the tumor mass and enter the bloodstream, and they originate either from primary sites or metastases that circulate freely in the peripheral blood. They have been detected in most epithelial cancers such as CRC (48). CTCs express similar markers as the cancer niche, and these markers are correlated with the occurrence of metastases and reduced survival in patients (49, 50). Furthermore, heterogeneous populations of CTC were detected as they demonstrated variants corresponding to different major regulatory pathways including KRAS, PIK3CA, and BRAF (51).

## Metabolism Characteristics of CSC That Enhance Invasiveness

The altered metabolism of CSCs causes them to fail in efficiently producing ATP, in part due to the hypoxic environment. Therefore, fulfillment of much of the energy requirements comes from glycolysis, which is the fastest energy-producing pathway (52–54) due to the Warburg effect that is characterized by high pyruvate and low lactate production (55–57). Furthermore, the high volume of glycolysis intermediates is then used in the pentose phosphate pathway (PPP) to produce ribose, which is necessary for nucleotide synthesis and in anabolic reactions for lipid synthesis (56, 58).

Interestingly, the Warburg effect and the enhancement of the PPP in CSCs seem to be responsible for the enhanced production of antioxidant molecules and the overall reduction of reactive oxygen species (ROS) demonstrated *in vivo* and *in vitro* models (15, 55, 59). CD44 (CD44v) expression in CSCs suppresses PKM2 (mice model), thereby decreasing pyruvate dehydrogenase activity and promoting PPP; these are all consistent with the stabilization of the cysteine/glutamate antiporter and elevated GSH levels (58, 60–62). All these lead to higher survival rates of CSC even in hostile environments (15, 57, 58). In isolated CRC, miR-124, miR-137, and miR-340 have been reported as being able to switch the Warburg effect's glycolytic mechanism into one involving oxidative phosphorylation in CRCs, which impairs cancer growth as a result (63). In the liver, miR-3662 suppresses the hypoxia-inducible factor (HIF)-1α-mediated Warburg effect in hepatocellular carcinomas, which results in an antitumor effect (64). Other research groups have reported several other miRs with different important observations regarding the Warburg effect. First, miR-145 reduces the Warburg effect by KLF4 silencing in bladder cancer (65), and second, in breast cancer, miR-548a-3p targets sine oculis homeobox 1 (SIX1), which is a transcription factor that causes the Warburg effect, and miR-30a-5p targets the lactate dehydrogenase A (LDHA)-mediated Warburg effect, thereby suppressing tumor growth and metastasis (66, 67). The aforementioned studies were done using animal models, hence showing the true potential of miRs in the biological system. Yet, we believe a CRC-focused study is essential, given the complexity and the tissue-dependent role of miRs.

In the liver, aldolase B, which is one of the three aldolase isoforms, participates in the interconversion of both fructose-1-phosphate and frutose-1,6-bisphosphate to dihydroxyacetone and glycerol-3-phosphate, which acts as a rate-limiting step for fructose metabolism in the liver (68–73). Moreover, aldolase A and aldolase B have been linked to EMT in colon cancer, as they seem to interact with HIF-1 (74). Moreover, another study in mice showed that metastatic CRC cells in the liver upregulate aldolase B (ALDOB), which then promotes fructose metabolism to fuel glycolysis, gluconeogenesis, and PPP, which is a mechanism that is not seen in primary sites (75).

CSCs also heavily rely on glutamine for their metabolic needs; this is supplemented by conversion of other circulating branched-chain amino acids to glutamate and further aminated to glutamine. It is known that glutamine metabolism regulates the sensitivity of CSCs to metformin via the AMPK-mTOR pathway (76). Furthermore, glutamine can be hydrolyzed to glutamate which can be used to produce GSH (60–62).

Therefore, CSCs can switch between glycolysis and oxidative phosphorylation (OXPHOS), but they also can be influenced by the cancer stroma microenvironment. For example, CAF-secreted metabolites including lactate and ketone bodies can lead to OXPHOS (77). Moreover, CSCs relying on OXPHOS have better use of their limited nutrients (52), and even when OXPHOS operates at a lower rate, it represents a more efficient source of ATP, providing a selective survival advantage to the CSCs (78).

Beyond energy production, mitochondria are also involved in cellular redox rate, ROS generation, calcium buffering, synthesis of intermediate molecules as acetyl-CoA and pyrimidines, apoptosis regulation, and immune modulation (52, 79). In contrast to other cancer cells, CSCs have enhanced mitochondrial activity, more ROS and higher rates of oxygen consumption, and an apoptosis deficit. Moreover, they depend on fatty acid oxidation of ATP and on NADH generation (77). Mitochondrial DNA (mtDNA) is a factor that regulates this metabolic flexibility due to it possessing genes encoding the electron transport chain; abnormalities in the mtDNA are associated with cancer progression and metastasis (79). Enhanced mitochondrial biogenesis is also a key factor for CSC functionality, whereas an increased mitochondrial mass can be used to identify cells with enhanced self-renewal capacity and chemoresistance (78).

A recent report on CRCs showed that cancer cells perform an alternative type of metabolism to reach the liver and establish secondary tumors. CRC cells induce the secretion of creatine kinase brain-type (CKB) into the microenvironment by downregulating miR-483 and miR-551a. CKB phosphorylates extracellular creatine to produce phosphocreatine, which is necessary to maintain cellular ATP levels. Moreover, the metastatic potential of cancer cells has been associated with the transcription co-activator peroxisome proliferator-activated receptor gamma co-activator 1 alpha (PGC-1α), which has been shown to couple oxygen consumption, OXPHOS, and mitochondrial biogenesis with the enhanced invasion characteristics of cancer cells (80).

Another metabolic stimulus derived from the TME is decreased extracellular pH levels, which are associated with CSC phenotypic features (e.g., slow-proliferating state, expression of stem cell markers, invasive capacities, and therapy resistance); this may also be involved in minimizing residual disease and long-term clinical dormancy/relapse (81). However, the transcriptional element sensitive to acidosis has not yet been reported. Decreased pH levels improve cell migration and invasion by enhancing the activity of matrix metallopeptidases and ECM remodeling (82). There have been clinical reports of pH effects on CRC liver metastasis, wherein acidic pH levels were associated with higher volumetric liver increase and a higher incidence of liver metastasis [62%] (83).

Hypoxia is a state characterized by low oxygen levels and, in cancer, is associated with changes in TME, leading to cancer progression. Under hypoxic conditions, CSCs express high levels of hypoxia-inducible factor 1α (HIF-1α), which in turn promote migration, invasion, and EMT (84). Upregulation of HIF-1α and HIF-2α under hypoxic conditions activates the change from OXPHOS to glycolysis through the expression of GLUT1 and glycolytic enzymes such as LDHA and pyruvate dehydrogenase kinase (PDH). However, a master regulator of gene expression in cancer cells under acidic conditions is HIF-2α, which acts through the activation of NAD^+^-dependent histone deacetylases (sirtuins) 1 and 6 (SIRT1/6), leading to the deacetylation of lysine residues in the HIF-2α regulatory amino-terminal transactivation domain (N-TAD) (85). In a rat liver colon cancer metastasis model, cells were injected into the portal vein, after which it was established that decreased pH significantly enhanced liver metastasis (83). By altering the states of hypoxia and levels of pH, we can see the influence on the energy balance of cells which is a crucial step in regulating invasiveness of pH regulator molecules such as monocarboxylate transporters, V-type H^+^ ATPases (V-ATPase), carbonic anhydrase (CA), Na^+^/H^+^ Exchanger (NHE), and Cl^−^/HCO3^−^ anion exchanger 2 play critical roles in maintaining pH homeostasis. Regulation of pH through miR-34a and miR-34c has been reported to regulate lactate dehydrogenase. Moreover, miR-24 and miR-224 play crucial roles in cancer progression by regulating the expression of their target pH regulatory molecules CAIX, Cl^−^/HCO3^−^ anion exchanger 1, and SLC4A4 (a Na^+^-coupled HCO^−3^ transporter), respectively. Furthermore, miR-494 expression in intestinal epithelial cells under acidic pH and lowered miR-224 expression in colorectal cancer are associated with methotrexate resistance (86).

Upregulation of miR-210 and miR-21 and downregulation of miR-126 expression are potential CRC biomarkers because of their participation in HIF-1α/VEGF signaling during the initiation of colon cancer. In a hypoxic microenvironment, achaete scute-like 2 (Ascl2) is overexpressed, leading to EMT by miR-200b repression while allowing regulatory feedback for CRC EMT-MET plasticity. Conversely, miR-199a downregulation has been associated with metastasis in CRC by enhancing HIF-1α/VEGF expression. In CRC, p53 downregulates miR-107, which, in turn, enhances HIF-1β expression. miR-107 overexpression reduces tumor growth, VEGF expression, and angiogenesis. Moreover, in CRC, miR-145 expression is reduced, which can regulate p70S6K1 expression, which, in turn, targets HIF-1-α and VEGF. miR-145 expression is negatively correlated with p70S6K1, which acts as a tumor suppressor in CRC (87).

In TME, hypoxia attracts tumor-associated macrophages (TAMs), which through IL-10 and IL-12 contribute to migration, invasion, and metastasis; moreover, adipocytes provide fatty acids as an energy source (88). Endothelial cells increase the expression of miR-210 via HIF-1α, which downregulates ephrin A3, resulting in decreased migration and decreased capillary-like endothelial cells (89). At the same time, HIF-1α contributes to autophagy and aerobic glycolysis, stimulating CAF, which contributes to tumor progression (84). Furthermore, hypoxia has been shown to increase the secretion of tumor-derived procollagen-lysine and 1-oxoglutarate 5-dioxygenase (PLOD1). PLOD1, which is an HIF target that hydroxylates lysine residues on collagen, promoting the formation of mature collagen crosslinks, which has been reported to facilitate metastasis (90). Moreover, it is worth noting that PLOD1 expression is upregulated in gastrointestinal cancer compared with normal tissues, predominantly in gastric cancer associated with the overexpression of p53 (91).

Under these conditions, CSC is more resistant to chemotherapy and radiotherapy because of the induction of HIF signaling and upregulation of stem pathways such as CD44 and Notch. Furthermore, CSC is more resistant because of lower ROS production. Moreover, CSC in a hypoxic microenvironment demonstrates immunosuppressive and immune evasion properties: for example, VEGF suppresses dendritic cell function and promotes the expression of PD1 ligand (PD-L1), which generates resistance to cell immunity. In clinical settings, the use of anti-PDL1 drugs is currently becoming an adopted strategy. Also, a downregulation of MHC complexes occurs that leads to immunosuppressive effects (92). Although HIF-1α expression is not associated with the primary lesion, it may be used as a prognostic marker in metastasis.

## Migrating CSC and Metastasis

**Figure 2:**
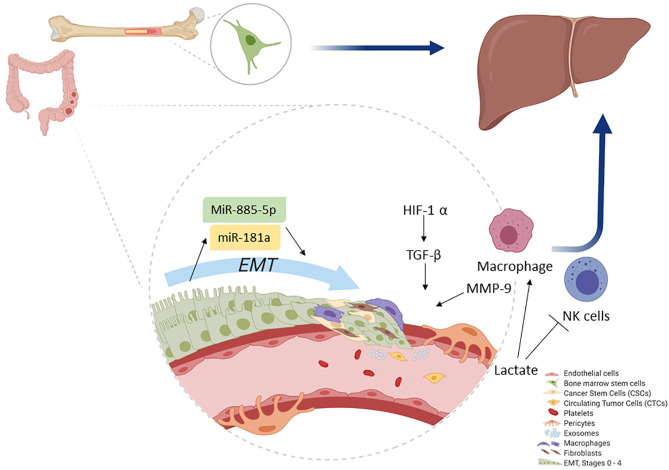
Colonocytes (from the primary tumor) undergo EMT. During EMT, initial activation of miR-181a leads, whereas further activity is accomplished by miR-885-50, thereby converting colonocytes to CSC. Macrophages play a key role as they help induce hypoxia and MMP activation, which ultimately leads to ECM removal. CSCs then convert to CTCs and migrate. The primary site for CTC migration is the liver; here, colon CTCs are further assisted by bone marrow stem cells to establish a new metastatic niche after which they invade.

The spread of colorectal CSC from the primary tumor to a secondary tumor requires phenotype changes such as the development of CTC. They require the formation of clusters together with stromal cells (e.g., fibroblasts, endothelial, tumor-infiltrated myeloid cells, or pericytes) to increase the viability of tumor progression, as shown in Figure 2. Pericytes promote the formation of CTC groups termed as circulating tumor microemboli, which are formed by 2–50 CTC with improved metastatic potential and tumor progression (15, 93).

As previously mentioned, certain CTC have all the hallmarks of CSC. They could develop metastasis, including the shedding and invasion of primary cancer cells into the circulatory system. They also have the abilities of migration, penetration of the vascular endothelial cell layer, tissue invasion, cell proliferation, and angiogenesis (93). Moreover, during tumor progression, some of the epithelial cells convert to CSC through EMT, thereby promoting angiogenesis. *In vitro*, these endothelial cells can transdifferentiate and acquire mesenchymal or myofibroblastic markers such as α-SMA and type I collagen with the stimulation of growth factors such as TGFβ, which leads to endothelial cytoskeleton remodeling and increased vessel permeability (94). Although there are other mechanisms of tumor dissemination and metastasis progression, we will focus on the most reported.

During cancer, EMT is regulated by extracellular stimuli derived from TME such as growth factors, cytokines, and physical stresses including hypoxia (45). Growth factors such as TGFβ can inhibit E-cadherin expression, which stimulates the relocation of the membrane in CRC during the invasion step (23). In detail, TGFβ production is stimulated by hypoxic stress from myeloid cells, mesenchymal cells, and CSC. Development of hypoxia activates genes encoding glucose transport, glycolytic enzymes, VEGF, PDGF, bFGF, erythropoietin, angiopoietin, and placental growth factor (95). Combined, these genes help promote angiogenesis and metastasis (90).

Recently, stem cell markers have been used to identify three CSC subpopulations within liver metastasis from CRC samples isolated from patients. One population within the tumor niche had the markers SOX2^+^/NANOG^+^/KLF4^+^/c-MYC^+^/OCT4^−^, and two populations in the peritumoral stroma had the markers SOX2^+^/NANOG^+^/KLF4^+^/c-MYC^+^/OCT4^−^ and SOX2^+^/NANOG^+^/KLF4^+^/c-MYC^+^/OCT4^+^. The presence of OCT4 in the stroma was theorized to represent the most primitive subpopulation of CSC. This report showed that a highly specialized microenvironment plays an important role in maintaining the stem cell pool in liver metastasis (96). Interestingly, this specialized microenvironment can express components of the renin–angiotensin system (PRR, ACE, ATIIR1, and ATIIR2) in a wide variety of cancers including CRC (97). Stromal cell-derived factor 1 (SDF-1) is another factor that upregulates E-cadherin expression. The process of E-cadherin redistribution on the cell surface indicates that EMT has a regulation that seems to be crucial in tumor spread; however, more analyses of epithelial status at different stages of the metastasis process is needed. Moreover, SDF-1 is expressed in stromal cells such as fibroblasts and endothelial cells *in vitro*, and its interaction with the CXCR4 receptor on the CSC improves CRC liver metastasis because of its chemoattractant role (98).

Pericytes and endothelial cells promote intravasation, which is the process wherein cancer cells move into the blood and lymphatic flow (15); however, tumor progress for angiogenesis due to the increased expression of metalloproteases and growth factors depends on the high expression of VEGF-A and Wnt7B, which are both expressed by macrophages that co-express Tie2. These macrophages mediate the loosening of vascular junction and enhance vascular permeability and MMP-9 expression by tumor-associated neutrophils. MMPs (MMP-2, MMP-3, MMP-7, MMP-9, MMP-13, and MT1-MMP) degrade the ECM, which then facilitates the extravasation of tumor cells and CSC (99). Endothelial cells, pericytes, and TAMs contribute to tumor intravasation by performing pro-angiogenic effects; conversely, VEGF, colony-stimulating factor-1 (CSF-1), and Ang2 induce neo-vessel formation via the expression of interleukin 8 (IL-8) (5, 100) in murine cancer models.

Not all CTC or CSC that undergo extravasation can generate metastases. Some CTC are not completely differentiated and have both epithelial cell markers such as epithelial CAM and mesenchymal cytokeratin markers such as cytokeratin (CK)-8, CK18, and CK19 (48). Subclones with an intermediate phenotype are known to have the greatest plasticity for microenvironment adaptation. These populations generate a more aggressive cell type with resistance to therapy and metastatic growth in breast cancer (101).

Aside from the difference between CTCs and primary tumor cells, heterogeneity also exists in different CTC subpopulations. Malara et al. showed different biological behaviors in two expanded CTC (eCTCs) subpopulations derived from patients with colon cancer. First, an eCTC subpopulation expressed CXCR4^+^CK20^+^; these were not tumorigenic cells. Next, a second eCTC subpopulation that additionally expressed CD45^−^CD133^+^ were tumorigenic. Patients with different CTC prevalence had different clinical outcomes (102). Thus, on the basis of this heterogeneous CTC composition, many researchers now believe that traditional clinical treatment strategies might not be useful for patients with metastasis, as these strategies are often based on the pathological and molecular characteristics of the primary tumor. This shows that CSC migration and metastasis are achieved by a combination of characteristics from cells; also, these microenviroments have been studied by different groups; nonetheless, CSC are capable of changing these properties to improve their survival and metastasis.

## Pre-Metastatic Niche

CTC invasion requires preparation of the microenvironment to allow for the development of metastasis (93). To establish the pre-metastatic niche, a series of contributors are necessary: (1) tumor-derived secreted factors, (2) extracellular vesicles, (3) immune cells, and (4) cell and molecular changes in distant organs that promote mobilization of bone marrow-derived cells and contribute to niche formation and tumor cell homing (103, 104).

In recent years, small non-coding RNAs such as microRNA (miR) have gained traction for their roles as regulatory factors in angiogenesis or in tumor metastasis during CRC (18, 104), as shown in Table 2. Other components of microenvironments involved in CRC progression to liver metastasis include immune cells such as CD11b^+^ myeloid cells that express integrins and various chemokines (CXCR2-CXCL1, CXCL12-CXCR4, and CCL12-CCR1) studied in a murine model of liver metastasis (126). Moreover, tumors and endothelial cells express sphingosine-1-phosphate receptor 1 (S1PR1) in a CRC murine model, which is a key element involved in the persistent activation of signal transducer and activator of transcription-3 (STAT3). STAT3 regulates inflammatory mediators (e.g., IL-6, IL-10, and TNFα, and GM-CSF) (127). Similarly involved are TAMs that release factors that contribute to tumor growth, immunotolerance, angiogenesis, and therapy resistance. M2 macrophages have protumor activity and are recruited in the metastatic and pre-metastatic niche by the cytokines CCL2, CCL5, VEGF, and CSF-1. *In vitro* studies have shown that in primary tumor, CAFs secrete fibronectin and lactate, which, when accumulated, have an inhibitory effect on natural killer (NK) cell activity while concomitantly promoting the expansion of M2 macrophages (Figure 2) (128, 129).

**Table 2:** miRNAs in premetastatic and metastatic niche.

	**miRNAs**	**Expression**	**Function in cancer**	**Target**	**References**
Pre-niche	miR-25-3p	Endothelial cells	Angiogenesis	KLF2 and KLF4	(105)
	miR-21^*^ miR-17^*^	Cancer cells Serum	Proliferation Invasion Cell infiltration	PDCD4PRL-3/STAT3	(106)
	miR-19	Cancer cells	An enzyme of ECM and TME	TG2	(107)
	miR-885-5p	Cancer cells	Migration, invasion	Cpeb2	(108)
	miR-20a-5p	Stromal cells	Invasion and metastasis	Smad4	(109)
	miRNA-155^*^	Cancer cells	Migration and invasion Lymph node metastasis	PTPRJ TP53INP1	(110)
	miR-181a	Cancer cells	Promotes EMT and liver metastasis	WIF-1	(111)
	miR-429	Cancer cells	Inhibits apoptosis Promotes EMT	HOXA5 SOX2	(112) (113)
Metastatic- niche	miR-527/665	CRC cells	Metastasis	KSRP SMAD4 TGFbR2	(114)
	miR-34a	Serum	Establishes metastasis	EGR1	(115)
	miR-99b-5p^*^	Liver metastasis	Inhibits rapamycin expression, tumor-suppressing miRNA, good prognostic	mTOR	(116)
	miR-377^*^	Liver metastasis	Lymph node metastasis, poor prognostic	p53, PTEN, and TIMP1	(116, 117)
	miR-200c, miR-196b-5p^*^	Liver metastasis, serum	Mediate epithelial-mesenchymal transition	TGF-TGFegu signal pathway	(116, 118, 119)
	miR-146a, miR-146a-5p	CRC cells	Migration and invasion	c-met, CPM (carboxypeptidase M)	(120, 121)
	miR-125, miR-127, miR-145, miR-194, and miR-199a-30^*^	stroma and tumor	Tumor suppressors, inhibit cell proliferation, motility, invasion, angiogenesis	c-Myc, KRAS, and MAPK4, BMP1, CDKN1B, mTOR, c-MET, ERK2,	(122, 123)
	Let-7a, miR-126, miR-141, and miR-21	Serum	Metastasis	KRAS, NIRF, IGFBP2, PITPNC1 MERTK ZEB1 and ZEB2 PTEN, Pdcd4, TPM1	(122, 124)
	miR-19a^*^, miR-19b, miR-23a, miR-92a, miR-320a and miR-4437	Serum	Inflammation, fibrosis, angiogenesis, liver metastasis, bad prognostic	TIA1 PTEN	(113, 123, 125)

* *Potential biomarker*.

**Figure 3:**
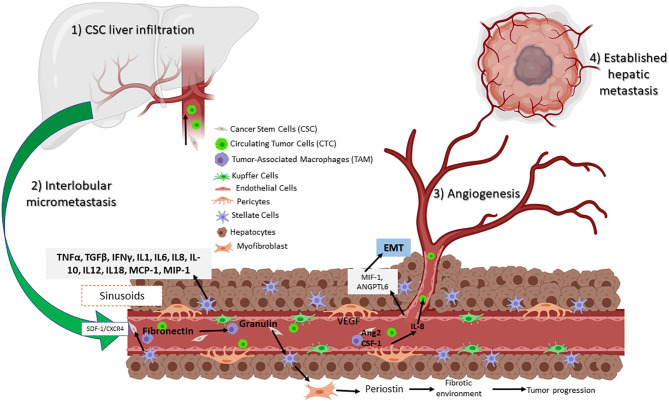
Circulating CSCs in the liver receive inflammatory signals such as VEGF and IL-8 from TAMs and pericytes. Signaling permits the conversion to CTC, which can then migrate and establish a new tumor niche. Assisted by hepatic stellar cells (HSCs) that transition to myofibroblasts, resulting in a fibrotic environment. Hepatic sinusoidal endothelial cells help to establish a new metastatic site.

Liver metastasis in CRC patients, which is not the only one presented, is most common because of the anatomical situation related to portal circulation (2). Once CSCs lose intercellular junctions in the primary tumor, they can diffuse through blood or lymphatic circulation and migrate into the portal system to the liver. They can traverse the endothelial barrier of the portal vessels, attach to hepatic sinusoids, and interact with sinusoid cells, Kupffer cells (KCs), hepatic stellate cells (HSCs), liver sinusoidal endothelial cells (LSECs), dendritic cells, liver-associated lymphocytes, and portal fibroblasts. ECM collagen and the cellular components of the liver adapt to the hypoxic environment, begin angiogenesis, and finally expand and metastasize. Metastatic lesions regularly respect the hepatic capsule and intersegmental layers, thus respecting nearby structures (130, 131); this process is illustrated in Figure 3.

Based on a large number of studies, liver metastasis progress is divided into four phases: (1) CSC liver infiltration, (2) interlobular micrometastasis, (3) angiogenesis, and (4) established hepatic metastasis (131). The first phase occurs within the sinusoids, whereas the following steps are further metastatic steps that affect the inner hepatic parenchyma. Moreover, resident cells generate multiple tumorigenic effects, promoting either elimination, CTC, or liver colonization. Here, a set of molecular pathways are involved including nitric oxide and ROS, as well as the expression of adhesion molecules such as selectins and integrins by the same cells. Also involved are phagocytosis, cytokines such as TNFα and TGFβ, interferon gamma (IFNγ), interleukins (IL-1, IL6, IL8, IL-10, IL12, and IL18), growth factor monocyte chemoattract protein-1 (MCP-1), and macrophage inflammatory protein (MIP-1) released by KCs and HSCs (132).

As mentioned before, macrophages are recruited to the liver transfer exosomes and to the KC, secreting TGFβ, which then promotes the production of fibronectin by HSC. When fibronectin accumulates, TAMs are recruited and form the pre-niche (Figure 3) (131). This was confirmed in a murine model of metastasic pancreatic ductal adenocarcinoma (PDAC). Expression of granulin by macrophages stimulates HSC for differentiation into myofibroblasts, which in turn releases periostin, creating a fibrotic environment in the liver that sustains tumor growth (133). Similarly, human hepatic sinusoidal endothelial cells *in vitro* express macrophage migration inhibitory factor (MIF-1), which induces EMT, migration, proliferation, and apoptotic resistance in CRC cells (134). Finally, angiopoietin-like 6 protein from the LSEC induces liver colonization of CRC cells and correlates with CRC progression in *in vitro* models (135).

The liver suffers different changes from correct establishment of metastasis, generated mainly due to CSC and its released factors. Moreover, other factors such as genetic mutations or epigenetic changes that improve CSC colonization in different tissue should be studied.

## Liver Metastasis and Colorectal Cancer

Once the microenvironment in the liver has been achieved, a series of changes occur in the cellular architecture that can lead to an inflammatory response (e.g., IL6-STAT3), which leads to metastasis in aggravated cases (136–138). The elevated expression of CD66e, which is also known as the carcinoembryonic antigen (CEA) or CEACAM5, is a common change because it is a widely expressed receptor in human carcinomas. Therefore, CEACAM5 is currently being evaluated as a determinant of the CRC state as its elevation is associated with metastasis (139, 140).

CEACAM5 affects the liver as follows: first, it protects circulating CRC cells from death through the inhibition of anoikis (an apoptosis mechanism in detached cells that occurs through a TRAIL-DR5 translocation). CEACAM5 binds the human RNA binding protein M4 in KC, and consequently blocks downstream translocation of DR5. Second, the bound form of CEACAM enhances the KC microenvironment by activating fibroblasts, which in turn facilitate CRC growth (139, 141). ECM expression of the α5β1 integrin/fibronectin interaction then leads to cell survival (142–144). Finally, CEACAM5 upregulates adhesion molecules such as E-cadherin and N-cadherin, thereby promoting aggregation (cell survival) and metastasis (139, 140, 144–146). Closely related is CD66c (or CEAMCAM6), which has also been implicated in reducing life expectancy, as it has been identified to be involved in similar signaling strategies (141, 147). A unique feature of CEACAM6 is its promotion of HER2 and TGFβ interactions. Overexpression of CEACAM6 increases SRC activity, leading to the enhancement of IGF-1 secretion resulting in MMP-2 and MMP-7 activation and ECM rearrangement (147). This knowledge has been demonstrated *in vitro* and clinical data; however, detailed mechanisms remain to be elucidated.

Increased accumulation of mutations in genes encoding downstream signaling pathways are well-studied drivers of CRC metastasis. Studies have shown that mutations in the MAPK/ERK pathway (KRAS), p53 pathway (TP53), Wnt/β catenin pathway (APC), and the PI3K/Atk pathway (PIK3CA) in clinical CRC samples from patients are the most influential drivers of liver metastasis. These have emerged as the most important biomarkers that determine the biological state of the tumor and provide clinical significance to patient outcomes (148–150).

The RAS family of proteins belongs to the guanosine-5′-triphosphatase family, among which KRAS is a well-studied member. Primarily functioning as a signal transducer, KRAS can work both as a positive and negative stimulator of the MAPK/ERK signaling pathway and is activated by ligands binding to RTKs (151). When activated, KRAS becomes adherent to the membrane by the prenylation of the CAAX domain with farnesyl transferase, subsequently inducing SOS1/2 phosphorylation. Primarily, activated RAS can signal either the PI3K or MAPK pathways. In CRC, KRAS activation has been associated with rapid and aggressive metastasis to the liver (150, 152). Further mutations such as KRASQ61, overamplify the effects of EGF even at low concentrations, whereas KRASG12 requires a translocation to become active, in all cases causing a positive feedback over the RAS-RAF-MEK-ERK axis (153) resulting in increased cell proliferation.

Moreover, IGF-1R (Insulin-like Growth Factor 1 Receptor) seems to increase during the anti-EGF state by immunotherapy. Both IGF-1R and STAT3 stimulate CRC development due to microenvironmental effects (6). CRC cells tested with monoclonal antibodies showed not only IGF-1R mRNA overexpression but also the overexpression of PI3K (153, 154). Interestingly, the PI3K/Akt pathway activates during EGF resistance, and its inhibition reverses resistance. A further IGF-1R knockdown blocks Akt activation, which is a known therapeutic target. Therefore, it is postulated that IGF-1R, as well as PIK3CA (the PI3K subunit), can induce secondary resistance to anti-EGFR therapy in CRC (153).

TP53 acquired mutations have been shown to affect a plethora of cancers including colon, stomach, breast, lung, brain, and esophageal cancers, and estimations indicate that over 50% of all cancer cases contain TP53 mutations (154–159). In CRC, missense mutations in the DNA binding domain lead to its oncogenicity. Several p53 amino acid hotspots such as R175, G245, R248, R249, R273, and R282 are known to induce “gain of function” properties. Moreover, these malignant mutations are correlated to a poor survival rate in patients; as such, these function as crucial survival biomarkers (160). These mutations are known to translocate with p63 (DNp63a) and p73, which accelerate TGFβ (increase RhoA activity) or the proto-oncogene BRAF (through EGF) metastasis (157, 161). A mechanism involving ΔNp63α (1 of 5 possible Tap64 isoforms related with oncogenic properties) activated by miR-527/665 repression suppresses the KSRP regulatory factor thus, miR-198 (a regulatory miR) switches off upregulating SMAD4 and TGFβR2 (114) *in-vitro* and *in-vivo*. In conclusion, in addition to the well-known role of TPs oncogenes, the microRNA network is largely implicated in cell development and upkeep, and is currently the focus of many research groups.

**Figure 4:**
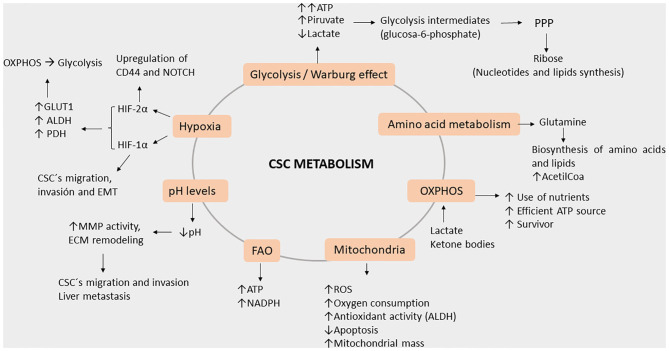
Roles of key processes in cancer stem cell metabolism during metastasis. Energy production occurs through the enhanced ATP production via glycolysis, amino acid metabolism (greater acetyl-CoA production), and OXPHOS (efficient use of nutrients). Furthermore, energy is also induced by hypoxia as HIF-1a and 2a (elevated GLUT1, ALDH, and PDH) both lead to OXPHOS and glycolysis. Additionally, potential damage to cells is reduced by NADPH as FAO activity and the Warburg effect increases, leading glycolytic intermediates to PPP. As hypoxia increases, the pH levels decrease, leading to MMP and ECM remodeling. Meanwhile, HIF-2a induces CD44 variants and the NOTCH signaling pathway, both activities acting in concert with decreased pH levels, thereby leading CSC to migration, invasion, and EMT.

Additionally, R248Q and R273C have been reported to bind and induce histone acetylation and increase HER2 protein levels, which is typically observed in 5–14% of RAS- and IGF-related CRC (147, 162, 163). Another important aspect of survival is the upregulation of NF-κβ and TNFα by blocking the effects of the tumor suppresor protein DAB2IP, as p53 mutants provide activation by increasing the production of cytokines IL-6, IL-11, and IL-23 (pro-angiogenic), as well as the production of cancer cells due to cytotoxic effects happening in the environment (Figure 4). Directed rearrangement to the microenvironment regulated by p53 mutants is exhibited as a high expression of CXCL5, CXCL8, and CXCL12, which endorses cells to migrate, grow, and invade and reshape antitumor mechanisms to its advantage as part of chronic tumor adaptation and growth (157).

APC mutants have been found in over 80% of CRC patients; additionally, hypermethylation has been described in nearly 20% of patients (164). Recently, a new player has emerged for its direct role in mediating functions of a mutated APC, namely, lncRNA-APC1. lncRNA (long non-coding RNA) APC1 is a downstream effector of APC and is independent of β-catenin (normally, it serves as a regulatory negative feedback product). It exerts strong antiangiogenic effect *in vivo* and in xenograft murine models by remarkably reducing exosome production through the Rab proteins (Ras mediates signaling). Rab5b levels seem to be upregulated during lncRNA-APC1 silencing in CRC, which, when further tested, has its silencing correlated to cell proliferation. Furthermore, subsequent testing demonstrated p38/MAPK, JNK, ATK, and Rho dependency on exosome production (165).

PIK3CA gene encodes for the p110α subunit of type I PI3Ka, and mutations in this gene are prevalent in ~30% of CRCs. Its primary function is to produce PIP3 as a way of inducing PDK1 and ATK signaling. PI3K expression combined with the loss of APC activity concludes in metastasis. It should be noted that both PI3K and APC mutants are strong biomarkers because their levels can help indicate a response to chemotherapy in preoperative patients (148).

As mentioned above, many aspects of liver metastasis have been revealed; however, better understanding of the biomarkers that have been reported as therapeutic targets is still required. There also exists the question: to what extent can the damage caused to the liver be reversible through therapeutic strategies? What we know is that out of the patients who undergo liver resectioning as a treatment, 75% have recurrence within the first 2 years after surgery that can be both intrahepatic and extrahepatic (146, 166). Therefore, new approaches that are specific against relevant cell populations, or are triggered by remaining cell types, are required to promote metastasis. Lastly, the underlying role of exosome-mediated extracellular communication has been progressively elucidated; its cargo, namely, proteins, transcription factors, growth factors, and several miRs (oncomirs) have been identified as important biomarkers, chemotherapy resistance, of prognostic value or very plausible therapeutic targets.

## CSC Potential Therapeutic Targets

Based on the various biological characteristics of CSCs, it is clear that they represent important targets against tumor progression and metastasis (6). Targeting CSCs via their unique signaling pathways (Sonic hedgehog (Shh)/Patched (Ptch)/Smoothened (Smo), Notch/Delta-like ligand (DLL), CXC chemokine receptor 1-2/CXCL8/FAK, Wnt, β-catenin, STAT3, and Nanog), metabolic reprogramming, targeting ABC transporters, and the use of non-coding RNA, represent promising strategies (167, 168). Napabucasin is a first-in-class cancer stemness inhibitor that targets STAT3. Furthermore, clinical evaluation of napabucasin was recently completed in a phase III clinical trial in CRC patients. Although no significant difference in overall survival was seen when comparing between the napabucasin and placebo treatment groups, in a subgroup of patients with pSTAT3-positive tumors, napabucasin treatment led to improved survival (169). However, because of the inherent heterogeneity of CSCs, targeting a single molecule or pathway may not be an effective strategy since CRC (and most cancers) rely on alternative pathways to avoid cancer checkpoints; therefore, encompassing most pathways may dictate better therapy; some of these are discussed later.

Immunotherapy represents an important emerging field in tumor therapy (170). A chimeric antigen receptor-modified T-cell (CAR-T) cocktail immunotherapy targeting CD133 and EGFR showed a longer partial response than single CAR-T target therapy in a patient with advanced CRC (171). Immune checkpoint inhibition is another immunotherapy strategy that has been recently applied to CRC. Programmed cell death protein 1 (PD-1) is expressed in T-cells, and PD-L1 is constitutively expressed in cancer cells. PD-L1/PD-1 interaction dysregulates T-cell activity, inducing exhaustion, apoptosis, neutralization, and promoting the production of IL-10, which in turn reduces cytotoxic T-cell (CD8+) activity, promoting cancer development and progression by enhancing tumor cell proliferation and survival (172). The PD-1/PD-L1 blockade has shown positive results in CRC patients with microsatellite instability-high or MMR-deficient (MSI-H) cancers, which is a type of CRC characterized by high somatic mutations (173). In 2017, both PD-1 inhibitors nivolumab and pembrolizumab were approved for the treatment of MSI-H CRC in the United States for treatment-refractory metastatic CRC (170). Pembrolizumab and nivolumad, under the commercial names of Keytruda and OPDIVO, respectively, have been approved by the FDA since 2014. Nevertheless, for better results, CRC immunotherapy requires a combination of PD-1/L1 and/or CTLA4 inhibition agents such as Nivolumab and Ipilimumab. Several TME factors may contribute to this therapy such as low T-cell infiltration, low number of type 1 T-helper cell activity, and low immune cytotoxicity (170).

Another approach of this type of CRC therapy is the use of vaccines with only peptides, peptide-expressing viruses, peptide-loaded antigen-presenting cells, or the application of peptide-specific T-cells that bind to human leukocyte antigen so that they can be recognized by T-cells for a specific antitumoral immune response. One of the first tumor-associated antigens ever identified was the CEA, which is also overexpressed in CRC as mentioned before (174). There are ongoing clinical trials of this type of immunotherapy in metastatic colorectal cancer (NCT03555149, NCT00154713, NCT02380443, and NCT04110093) in Phase II.

Although this is a very promising strategy because of it can regulate expression, the TME still plays a key role in progression; therefore, current treatments should focus on combining different strategies by attacking the primary tumor and metastasizing it against different cell targets. One of the most promising is the use of miR as therapeutic targets since these are specific and do not develop resistance. These are directed not only against CSCs (40) but also against pericytes and endothelial or inflammatory cells that promote tumor angiogenesis and metastasis.

As mentioned above, many aspects of liver CSC biology have been revealed through great efforts and contributions by various research groups. However, various physiological and mechanistic questions regarding liver CSCs remain to be elucidated. The current understanding of biomarkers has given us several tools to better diagnose and understand the future of prognosis in patients with CRC and liver metastasis. Known protein interactions, mutations, and other gene and protein identification techniques are at the forefront of current clinical usage (169).

## Conclusion

Many aspects of liver CSC biology have been revealed through great efforts, which have contributed in the elucidation of the important role of exosomes and miRs. Both of these are key components with proven roles in helping establish the pre-metastatic niche, participating in TME development, assisting in angiogenesis, and, most importantly, dictating micrometastasis regulation. Furthermore, both exosomes and miRs tend to favor pathways that induce chemoresistance in cells, as well those that aid cell invasion through EMT. However, various physiological and mechanistic questions of the roles CSCs play in liver metastasis remain to be elucidated, such as those regarding certain regulatory factors, which undoubtedly will be the next therapeutic targets in the study of metastasis.

Our current understanding of biomarkers has given us several tools to better diagnose and understand the future of prognosis in patients with CRC and liver metastasis. Known protein interactions, mutations, and other gene and protein identification techniques are at the forefront of modern-day clinical use. Currently at its early stages, research on miRs seems to have great potential as many miRs tend to be exported by exosomes. These miRs can be directly detected in serum, blood, urine, and other major fluids; hence, studies that combine multiple techniques and novel therapeutic approaches will improve the prognosis of liver metastasis in CRC patients.

## Author Contributions

AQ-R and EG-T performed literature analysis and wrote, discussed the revised manuscript, and designed the images. JI wrote, analyzed, and corrected the manuscript. CG-V performed literature analysis and wrote part of the text. All authors read and approved the final manuscript.

## Conflict of Interest

The authors declare that the research was conducted in the absence of any commercial or financial relationships that could be construed as a potential conflict of interest.
